# Supplementary material for: Quantifying absolute addressability in DNA origami with molecular resolution
Source: Nat Commun. 2018 Apr 23;9:1600. doi: 10.1038/s41467-018-04031-z (PMC5913233; doi:10.1038/s41467-018-04031-z)
Supplement: Supplementary file 1 — Supplementary Information [file 41467_2018_4031_MOESM1_ESM.pdf]

Supplementary Information

Quantifying Absolute Addressability in DNA origami with Molecular Resolution

Maximilian T. Strauss, Florian Schueder, Daniel Haas, Philipp C. Nickels, and Ralf Jungmann

**Supplementary Figure 1**	20-nm DNA origami grid structure
**Supplementary Figure 2**	caDNAno overview of the rectangular origami structure
**Supplementary Figure 3**	Overview of alignment procedure
**Supplementary Figure 4**	Interface of analysis software
**Supplementary Figure 5**	Overview image for Fig. 2
**Supplementary Figure 6**	Influence of Magnesium in folding buffer on staple detection
**Supplementary Figure 7**	Influence of storage conditions on detection values
**Supplementary Figure 8**	‘Arrow’-shaped DNA origami grid with 10 binding sites used in Fig. 3c
**Supplementary Figure 9**	Overview of DNA-PAINT images used for quantification in Fig. 3d
**Supplementary Figure 10**	Overview of DNA-PAINT images used for quantification in Fig. 3d
**Supplementary Figure 11**	Overview of 18 DNA origami structures used for the heatmap in Fig. 4
**Supplementary Figure 12**	Overview of DNA-PAINT images used for quantification in Fig. 4, Dataset 1
**Supplementary Figure 13**	Overview of DNA-PAINT images used for quantification in Fig. 4, Dataset 2
**Supplementary Figure 14**	Detection and incorporation heatmap
**Supplementary Figure 15**	CanDo simulation of RMS fluctuations
**Supplementary Figure 16**	Scaffold and staple detection experiments
**Supplementary Table 1**	Fit of Michaelis-Menten saturation curve
**Supplementary Table 2**	Overview of related studies and their incorporation values
**Supplementary Table 3**	Super-resolution data properties
**Supplementary Table 4**	Imaging conditions
**Supplementary Table 5**	Used DNA-PAINT sequences
**Supplementary Table 6**	List of core staples
**Supplementary Table 7**	List of biotinylated staples

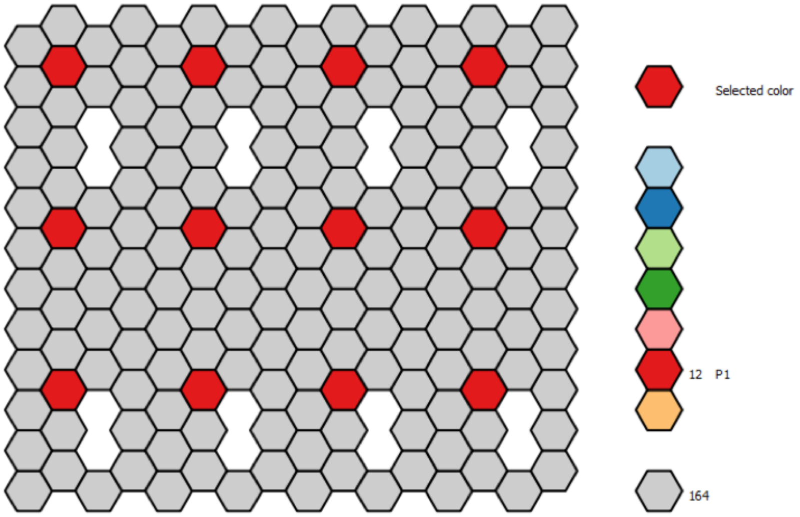

Supplementary Figure 1 | 20-nm DNA origami grid structure. Each hexagon represents a single staple, colored hexagons indicate a single staple extension at the 3'-end for DNA-PAINT probing.

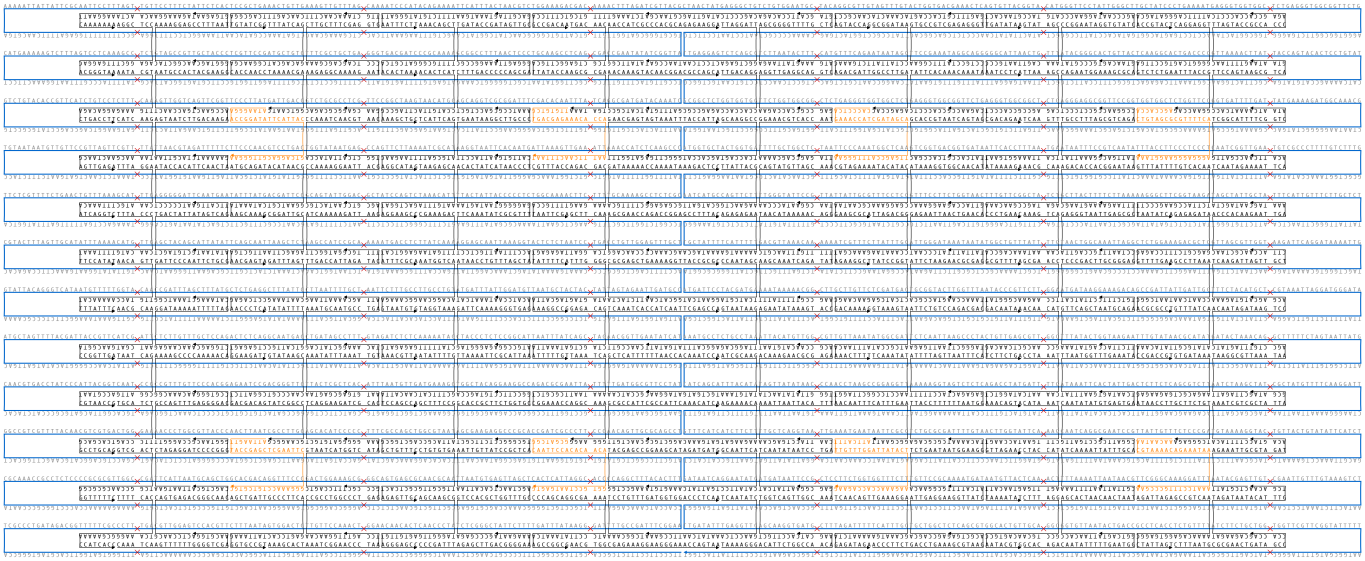

Supplementary Figure 2 | caDNAno overview of the rectangular origami structure. Blue: scaffold, black: unmodified staples, orange: biotinylated staples. For strand modification details see Supplementary Table 6.

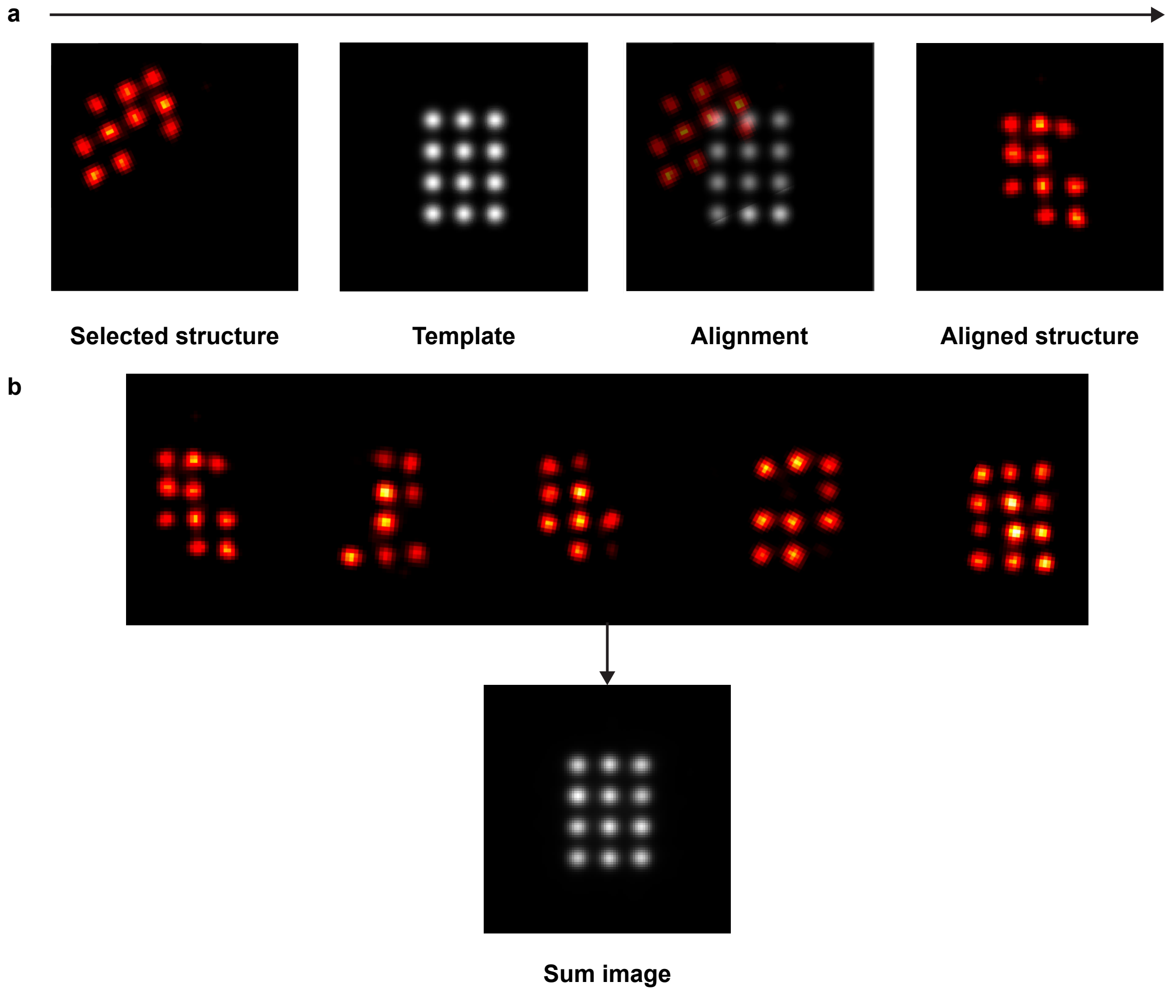

Supplementary Figure 3 | Overview of alignment procedure. **a**, DNA origami nanostructures are selected from the reconstructed DNA-PAINT super-resolution image. All selected structures are randomly orientated. A template is generated from the structure design. The selected structure is rotated stepwise and cross-correlated to the template. By selecting the angle where the correlation is maximised, the correct rotation angle can be obtained. The localisation data is rotated by the determined angle and corresponding shift, and the structure is aligned. **b**, Several aligned structures are stacked on top of each other to create a sum image which can be used for detection analysis.

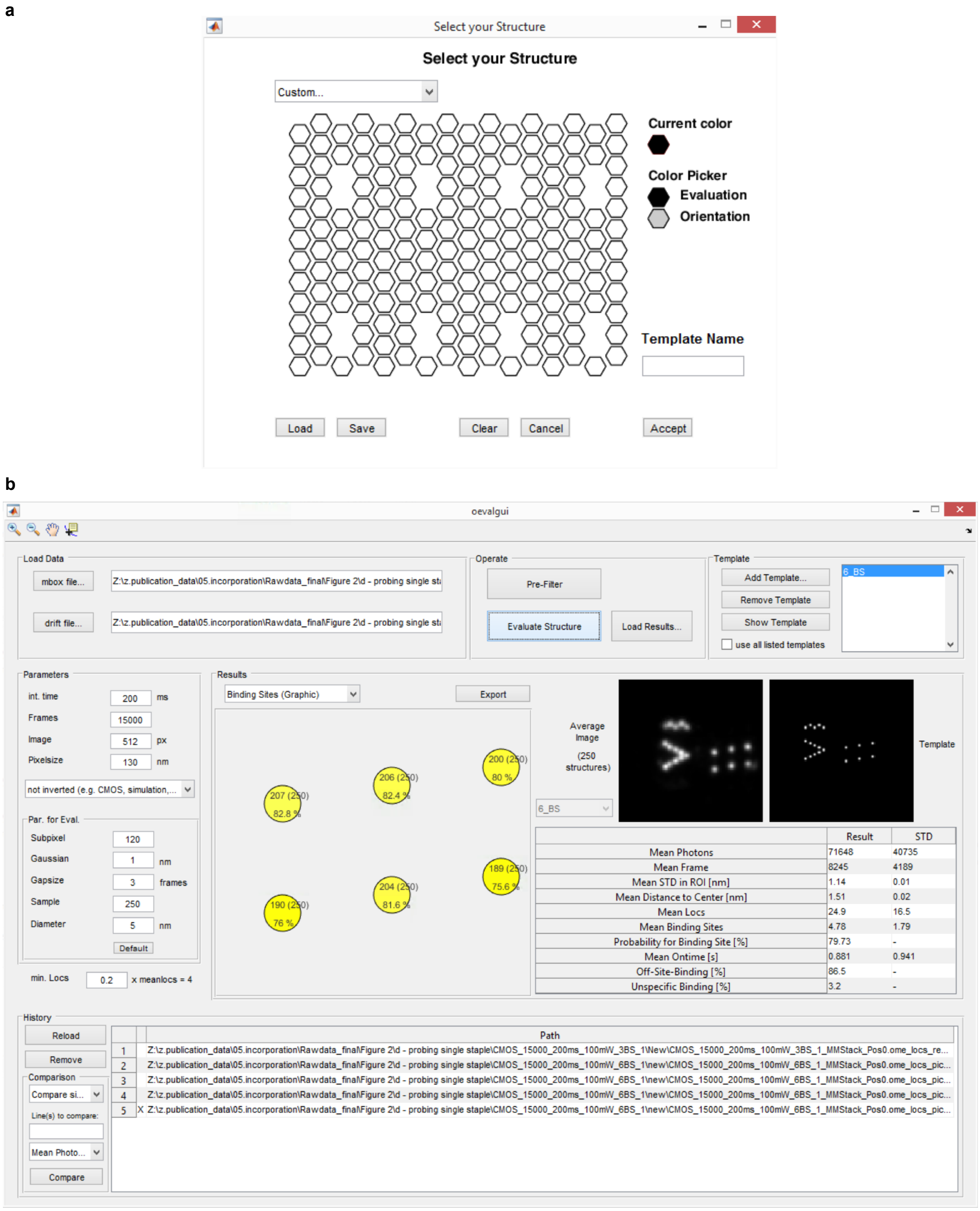

Supplementary Figure 4 | Interface of analysis software. **a**, Structure selection dialog. The analysis software features an interface where a structural template for the rectangular origami can be defined by clicking on hexagons. The software distinguishes between evaluation handles which will be evaluated and orientation handles which will be used in the template generation for the cross-correlation but not in the evaluation **b**, Main window. The main window features a display of the template, the sum image and detection statistics for the loaded dataset. Parameters for cross-correlation and thresholding can be set.

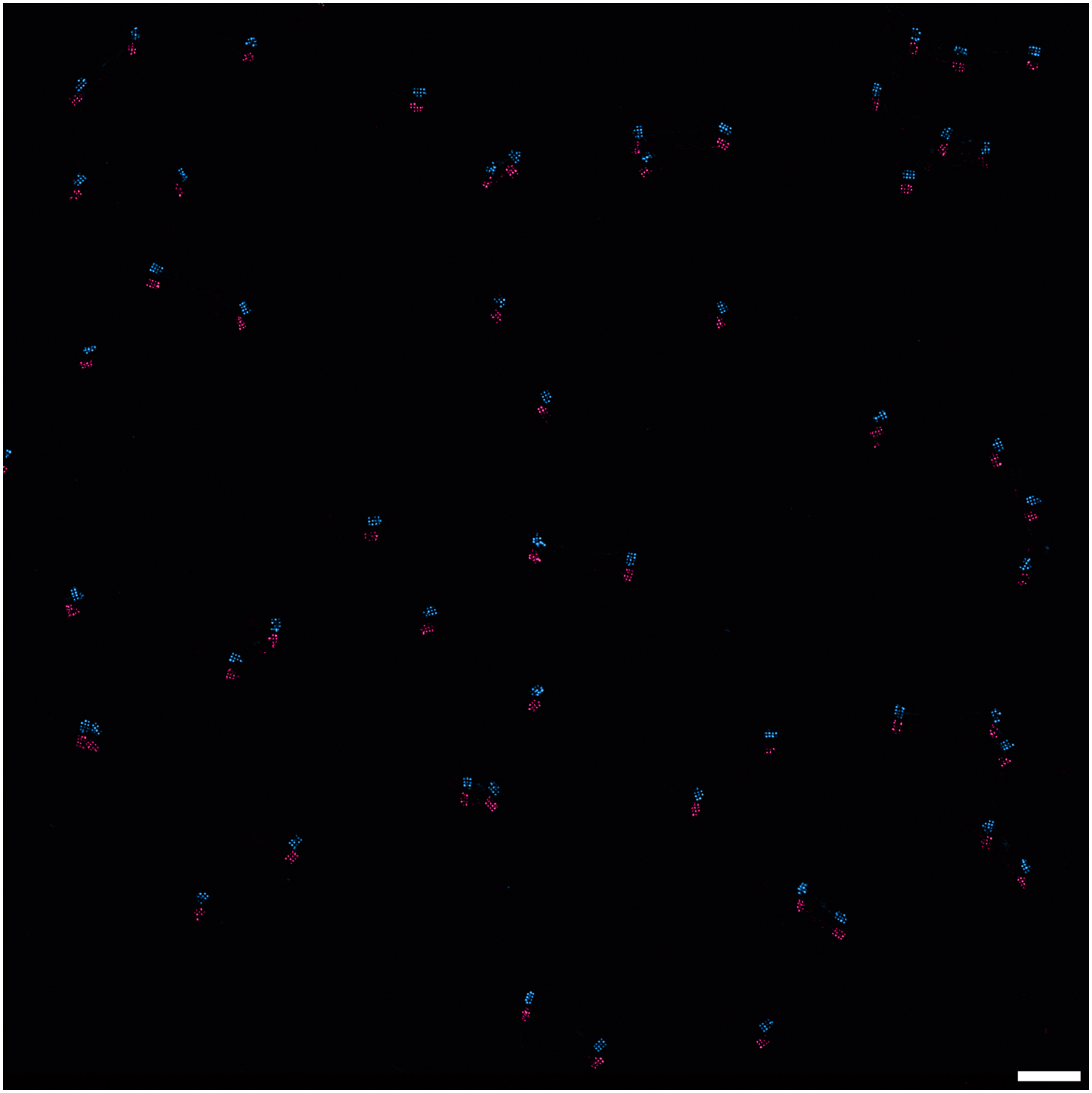

Supplementary Figure 5 | Overview image for Fig. 2. The two channels, red (Atto647N - 3’) and blue (Cy3b - 5’) are shifted to each other to allow better identification. Scale bar, 500 nm.

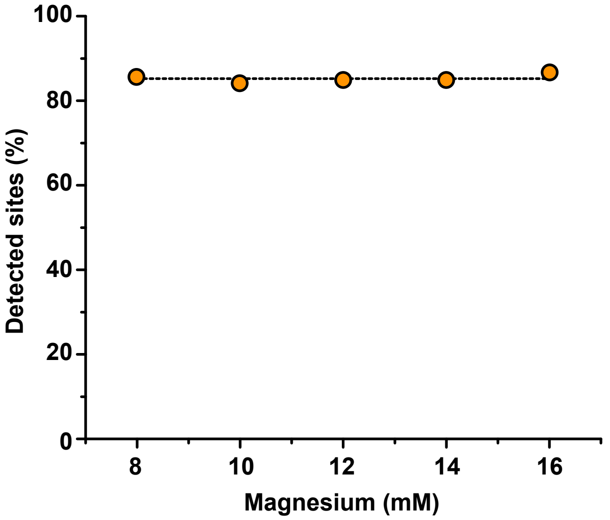

Supplementary Figure 6 | Influence of Magnesium in folding buffer on staple detection. DNA origami structures were folded at different Magnesium concentrations with a 3h folding ramp (60 to 4 °C). Modified staples had a 100× excess over Scaffold. Average detection (dashed line): 85.2%

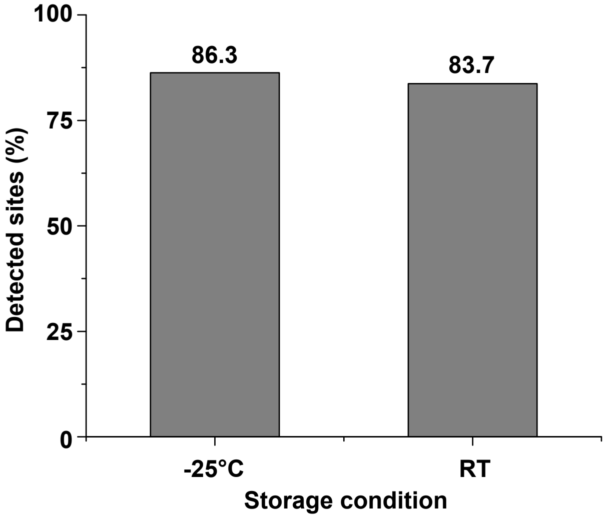

Supplementary Figure 7 | Influence of storage conditions on detection values. DNA origami structures were folded, PEG-purified and kept at room temperature and -25 °C for 58 days.

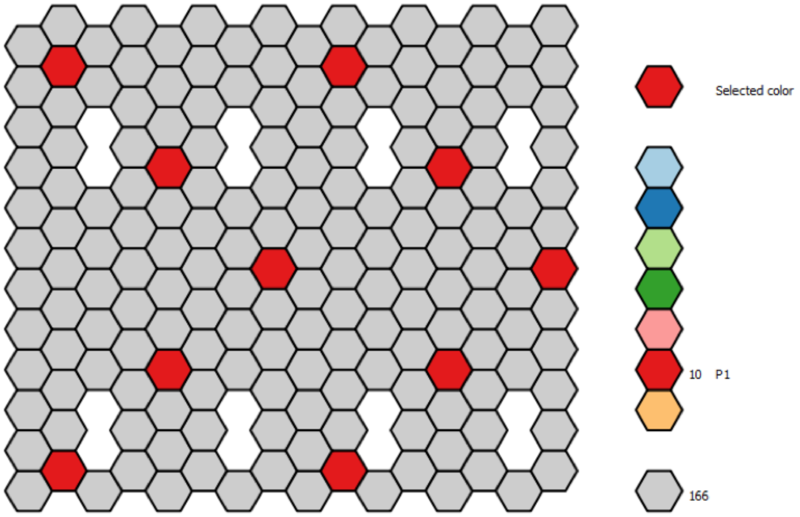

Supplementary Figure 8 | 'Arrow'-shaped DNA origami grid with 10 binding sites used in Fig. 3c Each hexagon represents a single staple, colored hexagons indicate a single staple extension at the 3'-end for DNA-PAINT probing.

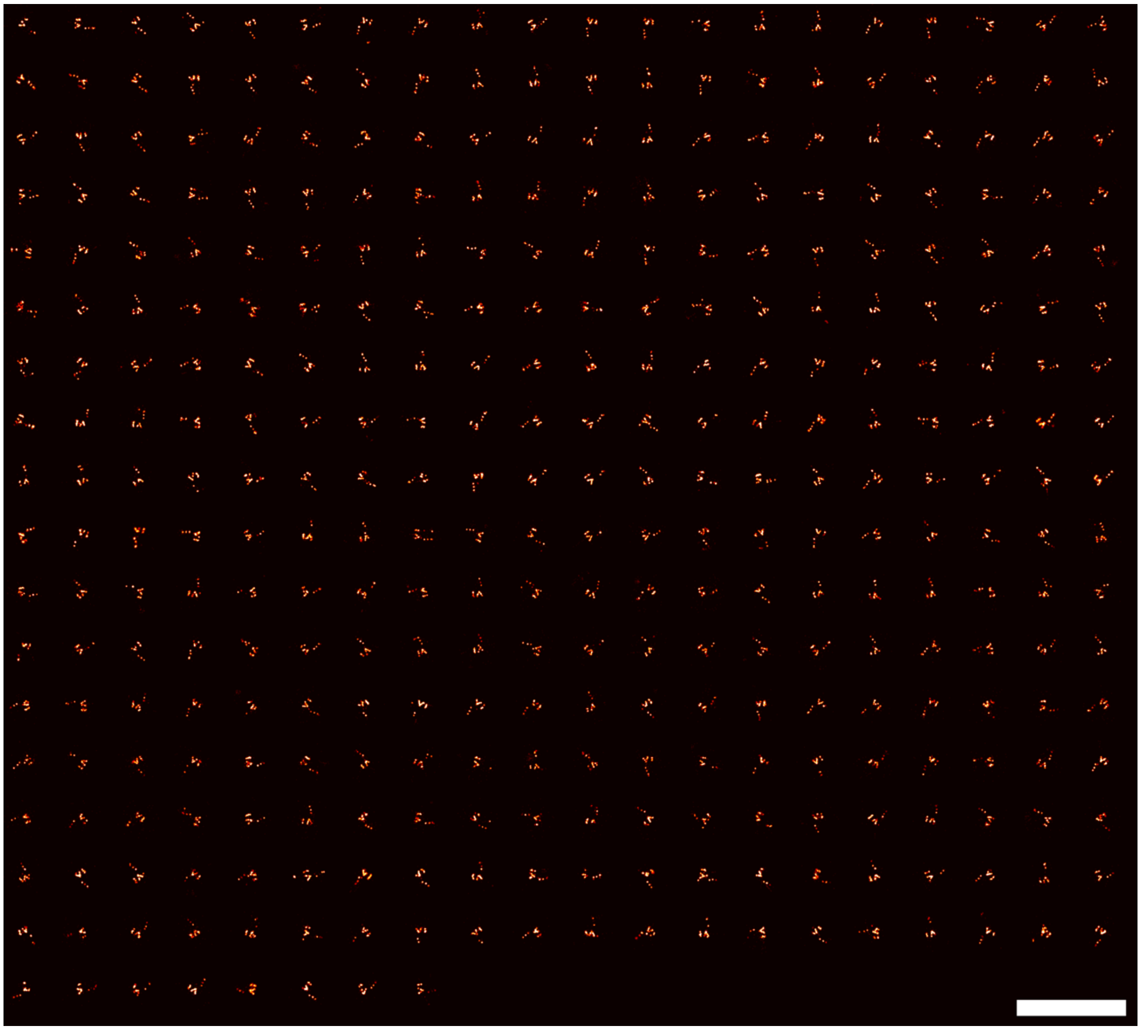

Supplementary Figure 9 | Overview of DNA-PAINT images used for quantification in Fig. 3d. 348 3BS structures are shown. Scale bar, 500 nm

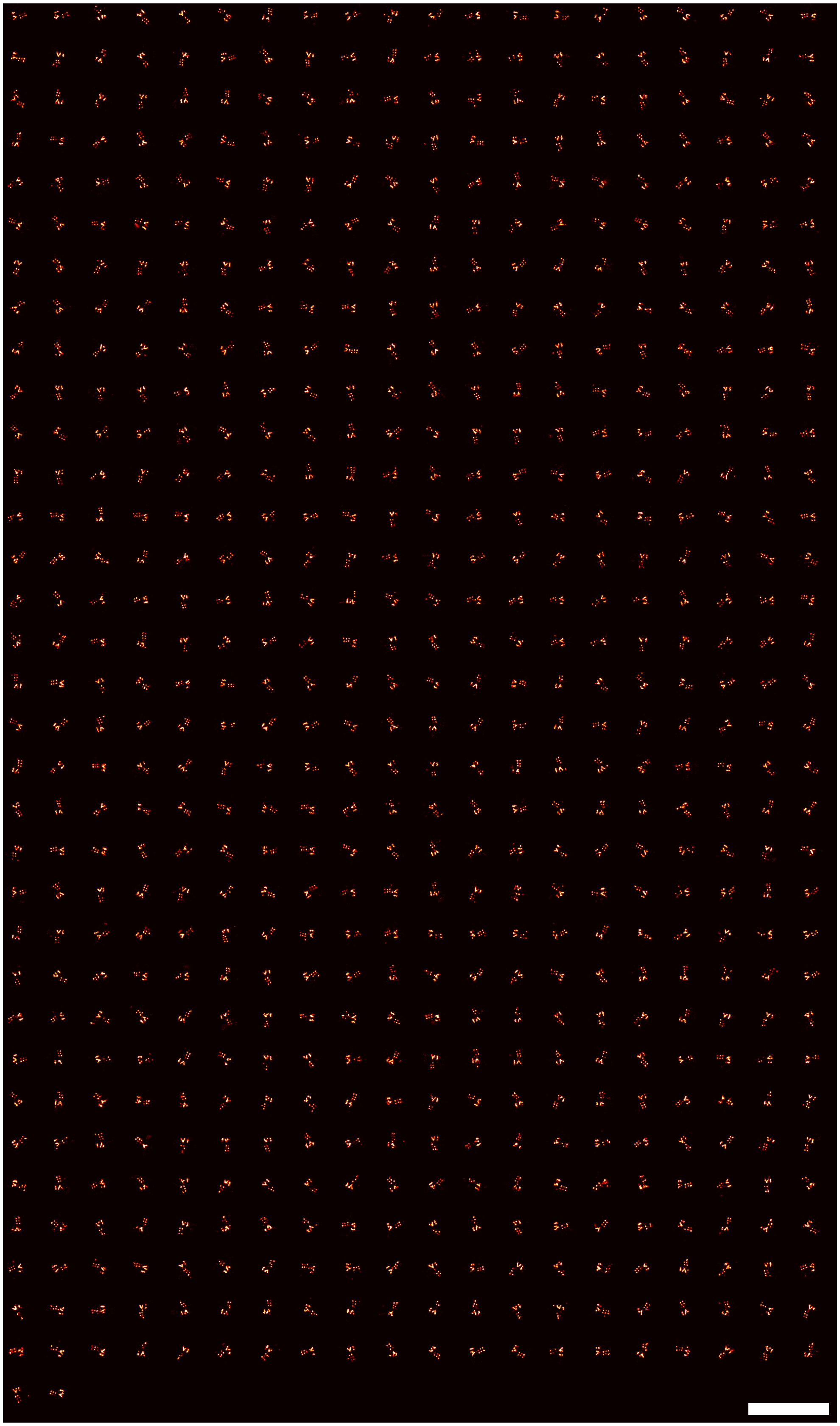

Supplementary Figure 10 | Overview of DNA-PAINT images used for quantification in Fig. 3d. 662 6BS structures are shown. Scale bar: 500 nm

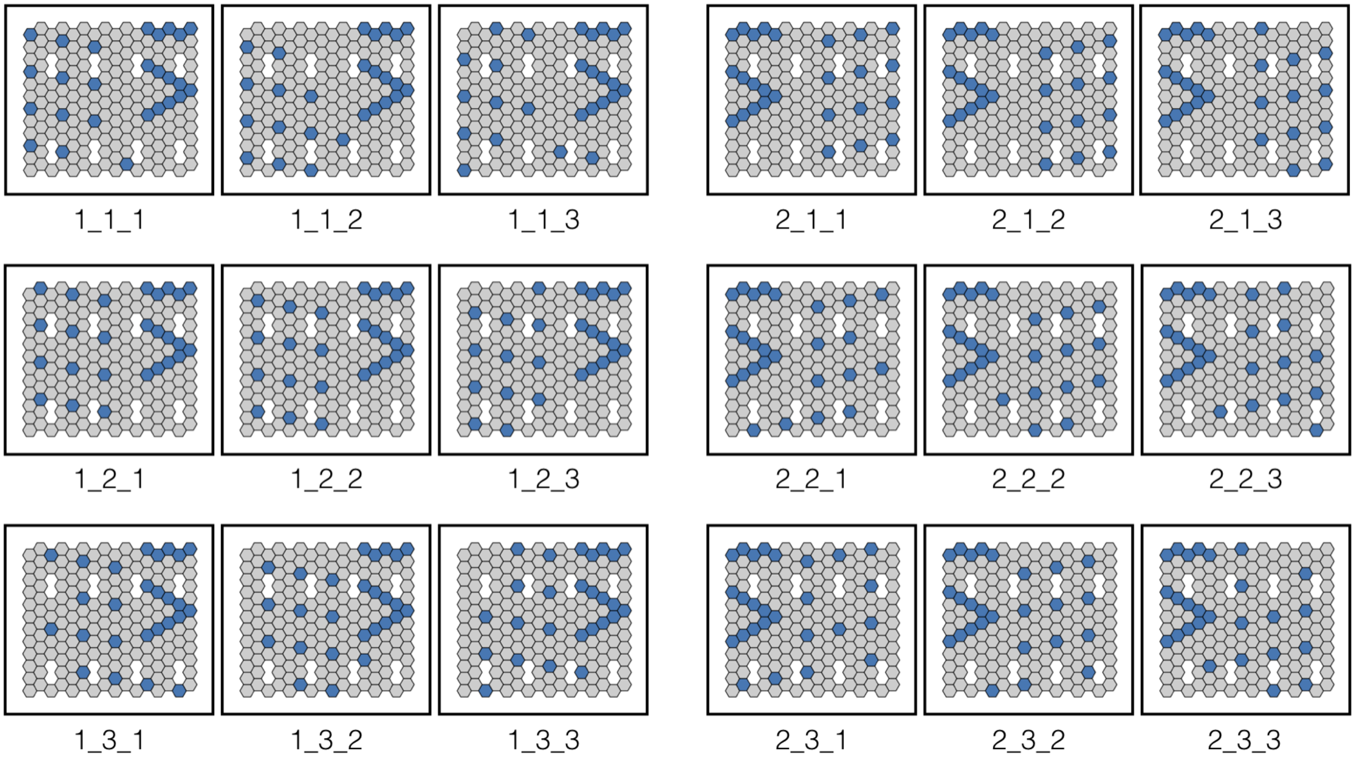

Supplementary Figure 11 | Overview of 18 DNA origami structures used for the heatmap in Fig. 4. The origami is divided into two sides, containing an orientation marker (arrow pattern) and staple positions to be probed.

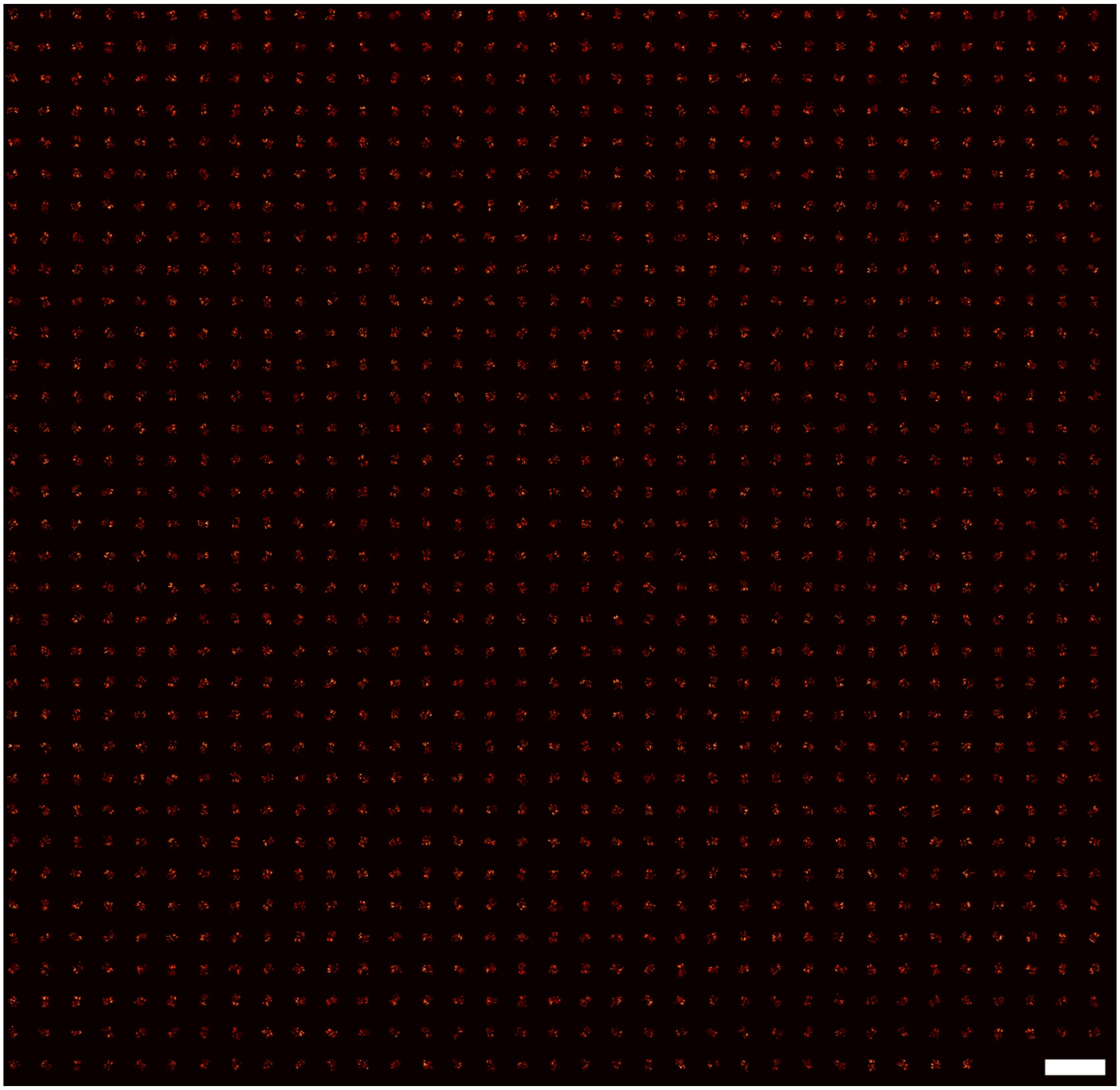

Supplementary Figure 12 | Overview of DNA-PAINT images used for quantification in Fig. 4, Dataset 1. 1186 structures are shown. The measurement contains all 18 origami structures. Scale bar: 500 nm

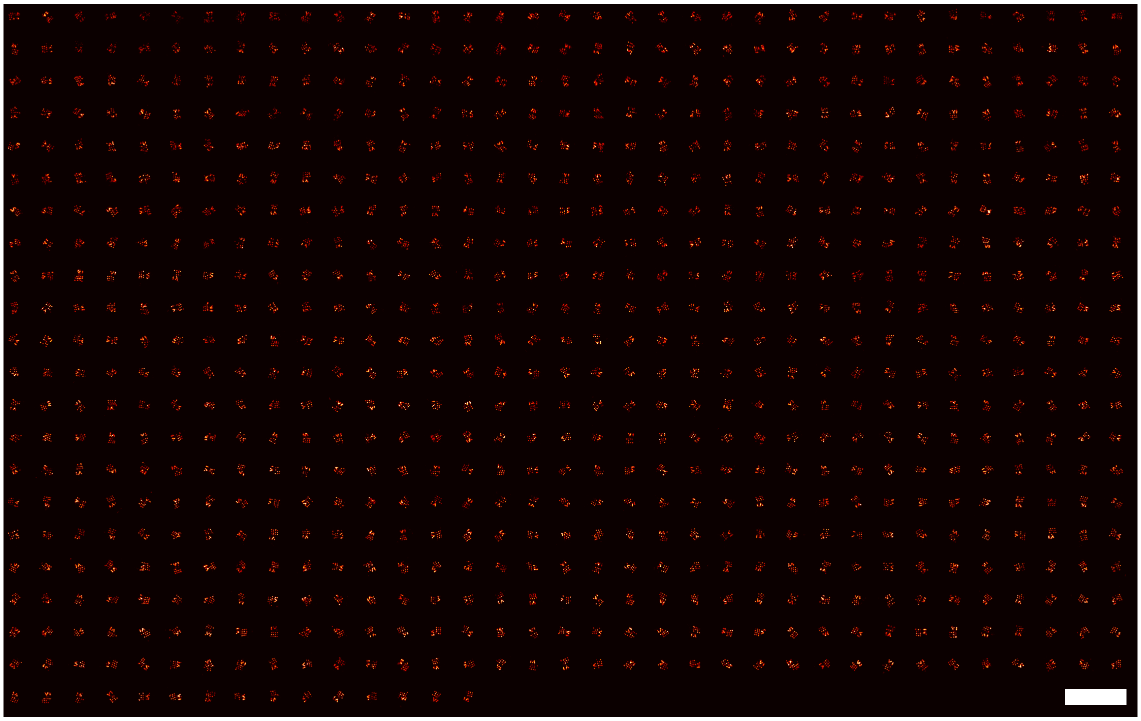

Supplementary Figure 13 | Overview of DNA-PAINT images used for quantification in Fig. 4, Dataset 2. 750 structures are shown. The measurement contains all 18 origami structures. Scale bar, 500 nm.

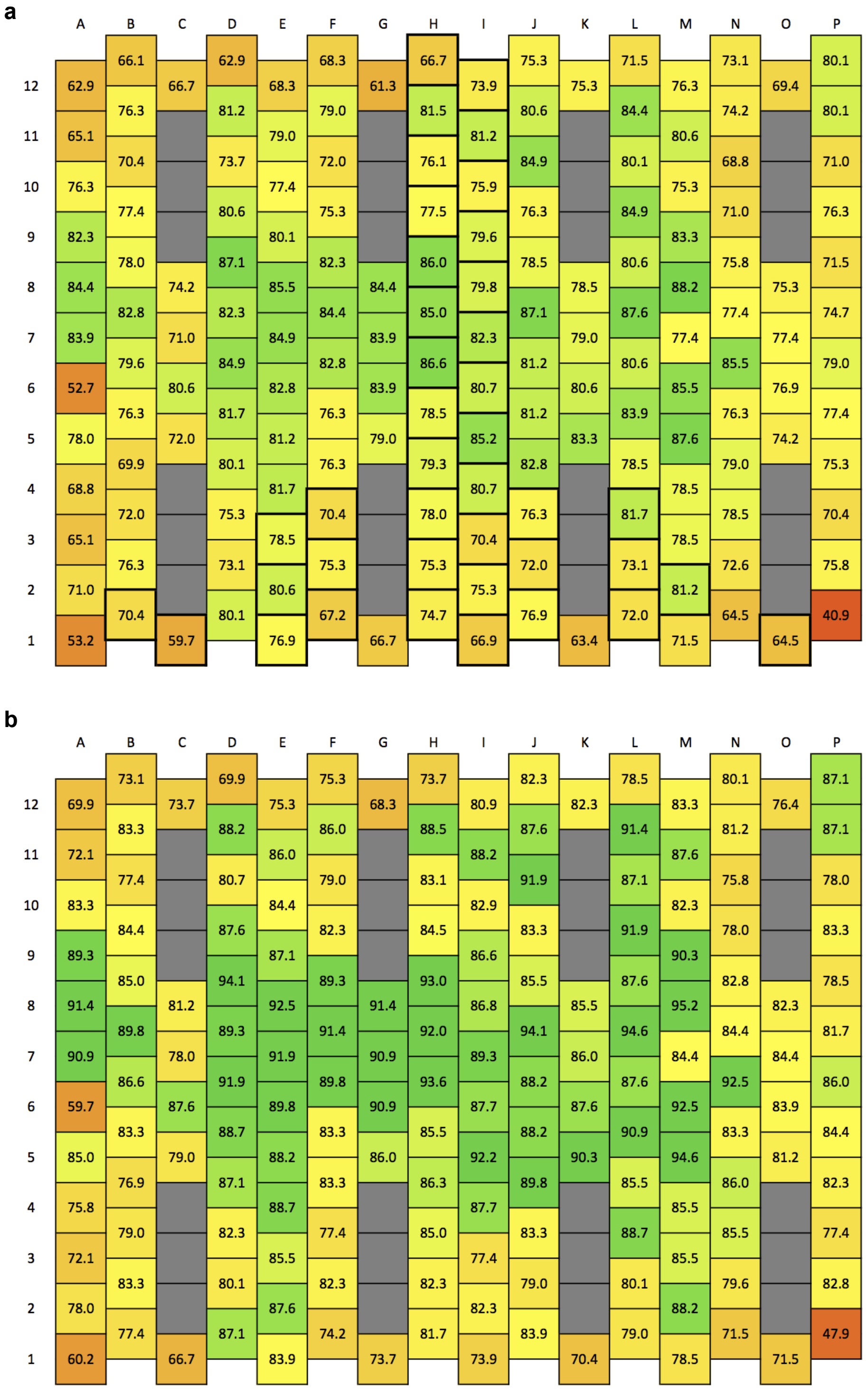

Supplementary Figure 14 | Detection and incorporation heatmap. a, The heatmap shows detection values for each probed position. **b**, The detection values can be translated to incorporation values by adding an offset of 7 % (as determined to be the 3'-error (see Figure 2) to generate an incorporation heatmap.

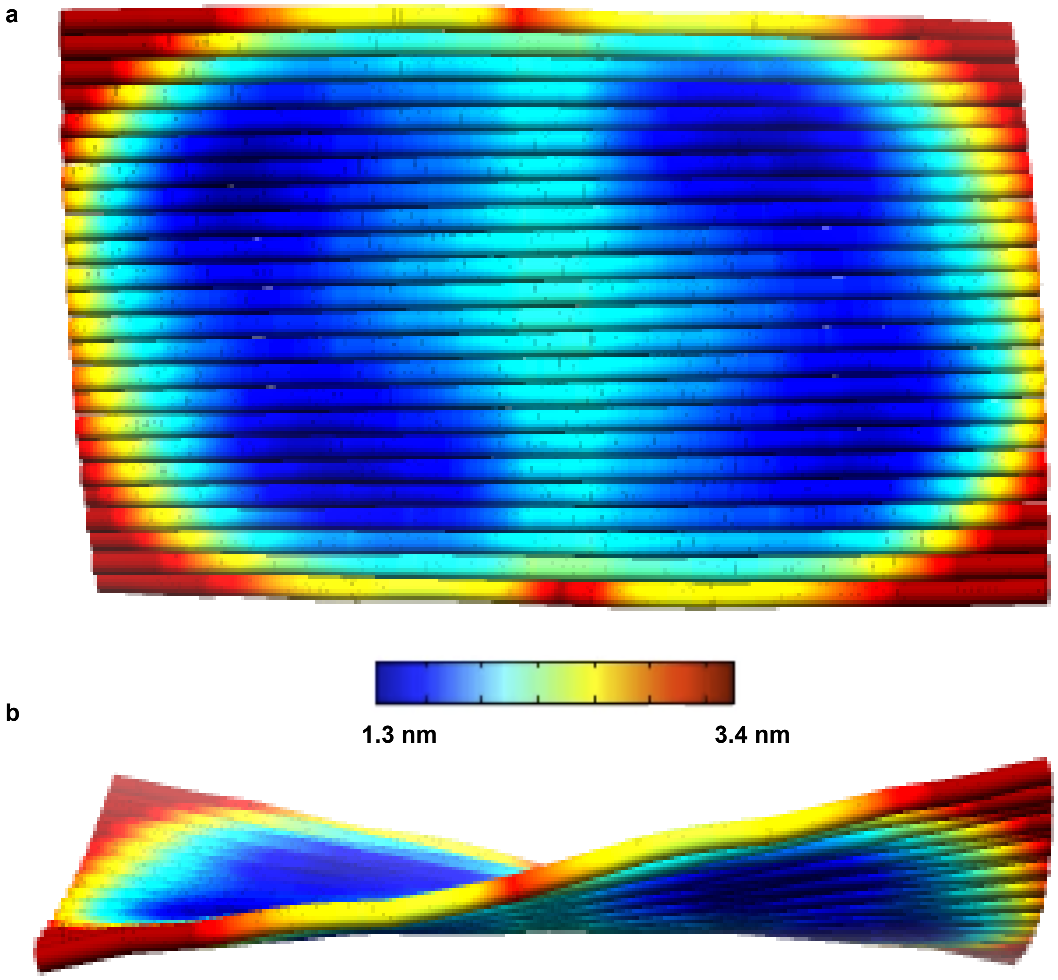

Supplementary Figure 15 | CanDo simulation of RMS fluctuations. a, Top view. The CanDo simulations show little thermal fluctuations in the center of the rectangular structure. Fluctuations with the highest magnitude can be found at the corners and edges. **b**, Side view. The side view shows the twisting of the DNA origami structure.

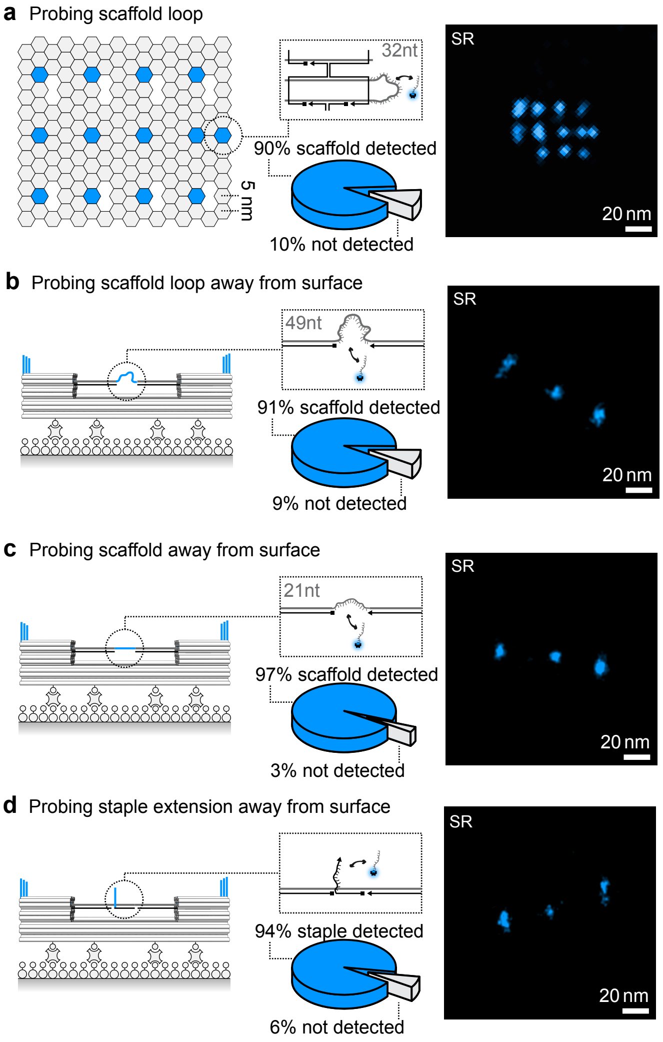

Supplementary Figure 16 | Scaffold and staple detection experiments. a, Probing the scaffold loop close to the surface yields a 90% detection efficiency. **b**, Probing a scaffold stretch (49 nt, similar to the loop of the flat origami) away from the surface yields a 91% detection efficiency. **c**, Probing a shorter scaffold stretch (21 nt, more ideally accessible) away from the surface yields a 97% detection efficiency **d**, Probing a staple extension away from the surface yields a 94% detection efficiency.

Supplementary Table 1 | Fit of Michaelis-Menten saturation curve

Name	Value	Standard Error
Formula	y = Vmax * x / (Km + x)	
Adj. R-Square	0.9805	
Vmax	83.13349	0.39929
Km	1.47056	0.11358

Supplementary Table 2 | Overview of related studies and their incorporation values. For (1) and (2) we estimated the incorporated for a single staple by considering the decay in 6 steps. For (1) this was from 100% to 74%, for (2) to 42%. For (3) we estimated the incorporation with the given probability of 98.7% that a particle is present being bound to three strands and backcalculation to one strand.

Reference	Structure and attachment	Incorporation
Jungmann et al. 2010	Streptavidin/Biotin	83%
Derr et al. 2012	Motor Protein	80%
Tomov et al. 2013	Hairpin Walker	95% (1)
Voigt et al. 2010	Streptavidin/Biotin	84%
Liber et al. 2015	Walker	86.50% (2)
Guer et al. 2016	Gold Nanoparticle Attachment	76.50% (3)
Jungmann et al. 2016	qPAINT	85%
Chatterjee et al. 2017	DNA Computing	92.5%/83%

Supplementary Table 3 | Super-resolution data properties

Measurement	NeNA
Fig 2: Atto647N	2.256 nm
Fig 2: Cy3b	1.616 nm
Fig 3: Annealing	10min: 1.573 nm<br>1h: 1.508 nm<br>3h: 1.664 nm<br>12h: 1.534 nm<br>3d: 1.768 nm
Fig 3: Excess	10x: 2.752 nm<br>20x: 2.144 nm<br>50x: 2.112 nm<br>100x: 2.72 nm<br>523x: 2.096 nm
Fig 3: 3 BS	4.017 nm
Fig 3: 6 BS	1.365 nm
Fig 4: Heatmap	1.495 nm / 1.469 nm

Supplementary Table 4 | Imaging conditions

Figure	Name	Origami	Excess	Folding Buffer	Folding Ramp	Purification	c(Origami)	Buffer	Imager	c(Img)-nM	Laserpower	Power density (kW/cm-2)	Int (ms)	Frames	Camera	ROI (px)
1 d	Simulations	20nm	-	-	-	-	50 structures	-	-	5		1.5	200	15000	160nm Px	128
2	5' - 3' Experiment	20nm	100	1x FB	80-4, 10min	Gel	17/100	B+/PPT	P1*	1		561 nm @ ~1.37	300	15000	EMCCD	256
2	5' - 3' Experiment	20nm	100	1x FB	80-4, 10min	Gel	17/100	B+/PPT	P9	1		640 nm @ ~1.97	300	15000	EMCCD	256
			100	1x FB	80-4, 10min	none	2/100	O+/PPT	P1	5	100	561 nm ~4.2	200	15000	sCMOS	512
			100	1x FB	80-4, 1h	none	2/100	O+/PPT	P1	5	100	561 nm ~4.2	200	15000	sCMOS	512
3	Folding Ramps	Arrow	100	1x FB	80-4, 3h	none	2/100	O+/PPT	P1	5	100	561 nm ~4.2	200	15000	sCMOS	512
			100	1x FB	80-4, 12h	none	2/100	O+/PPT	P1	5	100	561 nm ~4.2	200	15000	sCMOS	512
			100	1x FB	80-4, 3d	none	2/100	O+/PPT	P1	5	100	561 nm ~4.2	200	15000	sCMOS	512
			10	1x FB		PEG	3/100	O+	P1	5	70	561 nm @ ~2.9	300	7500	EMCCD	256
			20	1x FB		PEG	3/100	O+	P1	5	70	561 nm @ ~2.9	300	7500	EMCCD	256
3	Excess	20nm	50	1x FB	60to4,3h	PEG	2/100	O+	P1	5	70	561 nm @ ~2.9	300	7500	EMCCD	256
			100	1x FB		PEG	2/100	O+	P1	5	70	561 nm @ ~2.9	300	7500	EMCCD	256
			523.3	1x FB		PEG	2/100	O+	P1	5	70	561 nm @ ~2.9	300	7500	EMCCD	256
3d	Simulations	20nm	-	-	-	-	50 structures	-	-	5		1.5	200	15000	160nm Px	128
		1_1_1	100	1x FB		none										
		1_1_2	100	1x FB		none										
		1_1_3	100	1x FB		none										
		1_2_1	100	1x FB		none										
		1_2_2	100	1x FB		none										
		1_2_3	100	1x FB		none										
		1_3_1	100	1x FB		none										
		1_3_2	100	1x FB		none										
4	Heatmap	1_3_3	100	1x FB	60to4,3h	none	18x 0.2/100	O+/PPT	P1	2	100	561 nm @ ~4.2	###	##	CMOS	###
		2_1_1	100	1x FB		none										
		2_1_2	100	1x FB		none										
		2_1_3	100	1x FB		none										
		2_2_1	100	1x FB		none										
		2_2_2	100	1x FB		none										
		2_2_3	100	1x FB		none										
		2_3_1	100	1x FB		none										
		2_3_2	100	1x FB		none										
		2_3_3	100	1x FB		none										
			100	1x FB (8mM $MgCl_2$)		none	2/100	O+/PPT	P1	5	100	561 nm ~4.2	200	15000	sCMOS	512
			100	1x FB (10mM $MgCl_2$)		none	2/100	O+/PPT	P1	5	100	561 nm ~4.2	200	15000	sCMOS	512
SI	Magnesium	Arrow	100	1x FB (12mM $MgCl_2$)	60to4,3h	none	2/100	O+/PPT	P1	5	100	561 nm ~4.2	200	15000	sCMOS	512
			100	1x FB (14mM $MgCl_2$)		none	2/100	O+/PPT	P1	5	100	561 nm ~4.2	200	15000	sCMOS	512
			100	1x FB (16mM $MgCl_2$)		none	2/100	O+/PPT	P1	5	100	561 nm ~4.2	200	15000	sCMOS	512
SI 16	Scaffold Loop (a)	20nm	100	1x FB	80-4, 10min	Gel	17/100	B+/PPT	P9	1		640 nm @ ~1.97	300	15000	EMCCD	256
	Force Clamp (b,c,d)	Force Clamp	100	1x FB	Refer to Paper	Gel	500pM	B+/PPT	P1	5	70	561 nm @ ~2.9	200	15000	sCMOS	512

Supplementary Table 5 | Used DNA-PAINT sequences

Shortname	Docking sequence	Imager sequence	Experiment
P1	TT ATACATCTA	CTAGATGTAT-Cy3b	Fig 3/4
P1*	TT ATACATCTA	Cy3b-CATCCTAATT	Fig 2
P9	TT AATTAGGAT	CATCCTAATT-Atto647N	Fig 2

Supplementary Table 6 | List of core staples

Position	Name	Sequence
A1	21[32]23[31]BLK	TTTTCACTCAAAGGGCGAAAAACCATCACC
B1	23[32]22[48]BLK	CAAATCAAGTTTTTTGGGGTCGAAACGTGGA
C1	21[56]23[63]BLK	AGCTGATTGCCCTTCAGAGTCCACTATTAAAGGGTGCCGT
D1	23[64]22[80]BLK	AAAGCACTAAATCGGAACCCTAATCCAGTT
E1	21[96]23[95]BLK	AGCAAGCGTAGGGTTGAGTGTTGTAGGGAGCC

F1	23[96]22[112]BLK	CCCGATTTAGAGCTTGACGGGGAAAAAGAATA
G1	21[120]23[127]BLK	CCCAGCAGGCGAAAAATCCCTTATAAATCAAGCCGGCG
H1	21[160]22[144]BLK	TCAATATCGAACCTCAAATATCAATTCCGAAA
I1	23[128]23[159]BLK	AACGTGGCGAGAAAGGAAGGGAAACCAGTAA
J1	23[160]22[176]BLK	TAAAAGGGACATTCTGGCCAACAAAGCATC
K1	21[184]23[191]BLK	TCAACAGTTGAAAGGAGCAAATGAAAAATCTAGAGATAGA
L1	23[192]22[208]BLK	ACCCTTCTGACCTGAAAGCGTAAGACGCTGAG
M1	21[224]23[223]BLK	CTTTAGGGCCTGCAACAGTGCCAATACGTG
N1	23[224]22[240]BLK	GCACAGACAATATTTTTGAATGGGGTCAGTA
O1	21[248]23[255]BLK	AGATTAGAGCCGTCAAAAAACAGAGGTGAGGCCTATTAGT
P1	23[256]22[272]BLK	CTTTAATGCGCGAACTGATAGCCCCACCAG
A2	19[32]21[31]BLK	GTCGACTTCGGCCAACGCGCGGGGTTTTTC
B2	22[47]20[48]BLK	CTCCAACGCAGTGAGACGGGCAACCAGCTGCA
D2	22[79]20[80]BLK	TGGAACAACCGCCTGGCCCTGAGGCCCGCT
E2	19[96]21[95]BLK	CTGTGTGATTGCGTTGCGCTCACTAGAGTTGC
F2	22[111]20[112]BLK	GCCCGAGAGTCCACGCTGGTTTGCAGCTAACT
H2	19[160]20[144]BLK	GCAATTCACATATTCCTGATTATCAAAGTGTA
I2	22[143]21[159]BLK	TCGGCAAATCCTGTTTGATGGTGGACCCTCAA
J2	22[175]20[176]BLK	ACCTTGCTTGGTCAGTTGGCAAAGAGCGGA
L2	22[207]20[208]BLK	AGCCAGCAATTGAGGAAGGTTATCATCATTTT
M2	19[224]21[223]BLK	CTACCATAGTTTGAGTAACATTTAAAATAT
N2	22[239]20[240]BLK	TTAACACCAGCACTAACAACTAATCGTTATTA
P2	22[271]20[272]BLK	CAGAAGATTAGATAATACATTTGTCGACAA
A3	17[32]19[31]BLK	TGCATCTTTCCCAGTCACGACGGCCTGCAG
B3	20[47]18[48]BLK	TTAATGAACTAGAGGATCCCCGGGGGGTAACG
D3	20[79]18[80]BLK	TTCCAGTCGTAATCATGGTCATAAAAGGGG
E3	17[96]19[95]BLK	GCTTTCCGATTACGCCAGCTGGCGGCTGTTTC
F3	20[111]18[112]BLK	CACATTAAAATTGTTATCCGCTCATGCGGGCC
H3	17[160]18[144]BLK	AGAAAACAAAGAAGATGATGAAACAGGCTGCG
I3	20[143]19[159]BLK	AAGCCTGGTACGAGCCGGAAGCATAGATGATG
J3	20[175]18[176]BLK	ATTATCATTCAATATAATCCTGACAATTAC
L3	20[207]18[208]BLK	GCGGAACATCTGAATAATGGAAGGTACAAAAT
M3	17[224]19[223]BLK	CATAAATCTTTGAATACCAAGTGTTAGAAC
N3	20[239]18[240]BLK	ATTTTAAAATCAAAATTATTTGCACGGATTCG
P3	20[271]18[272]BLK	CTCGTATTAGAAATTGCGTAGATACAGTAC
A4	15[32]17[31]BLK	TAATCAGCGGATTGACCGTAATCGTAACCG
B4	18[47]16[48]BLK	CCAGGGTTGCCAGTTTGAGGGGACCCGTGGGA
C4	15[64]18[64]BLK	GTATAAGCCAACCCGTCGGATTCTGACGACAGTATCGGCCGCAAGGCG

D4	18[79]16[80]BLK	GATGTGCTTCAGGAAGATCGCACAATGTGA
E4	15[96]17[95]BLK	ATATTTTGGCTTTCATCAACATTATCCAGCCA
F4	18[111]16[112]BLK	TCTTCGCTGCACCGCTTCTGGTGCGGCCTTCC
G4	15[128]18[128]BLK	TAAATCAAAATAATTCGCGTCTCGGAAACCAGGCAAAGGGAAGG
H4	15[160]16[144]BLK	ATCGCAAGTATGTAAATGCTGATGATAGGAAC
I4	18[143]17[159]BLK	CAACTGTTGCGCCATTCGCCATTCAAACATCA
J4	18[175]16[176]BLK	CTGAGCAAAAATTAATTACATTTTGGGTTA
K4	15[192]18[192]BLK	TCAAATATAACCTCCGGCTTAGGTAACAATTTCATTTGAAGGCGAATT
L4	18[207]16[208]BLK	CGCGCAGATTACCTTTTTTAATGGGAGAGACT
M4	15[224]17[223]BLK	CCTAAATCAAAATCATAGGTCTAAACAGTA
N4	18[239]16[240]BLK	CCTGATTGCAATATATGTGAGTGATCAATAGT
O4	15[256]18[256]BLK	GTGATAAAAAGACGCTGAGAAGAGATAACCTTGCTTCTGTTCGGGAGA
P4	18[271]16[272]BLK	CTTTTACAAAATCGTCGCTATTAGCGATAG
A5	13[32]15[31]BLK	AACGCAAAATCGATGAACGGTACCGGTTGA
B5	16[47]14[48]BLK	ACAAACGGAAAAGCCCCAAAAACACTGGAGCA
C5	13[64]15[63]BLK	TATATTTTGTCATTGCCTGAGAGTGGAAGATT
D5	16[79]14[80]BLK	GCGAGTAAAAATATTTAAATTGTTACAAAG
E5	13[96]15[95]BLK	TAGGTAAACTATTTTTGAGAGATCAAACGTTA
F5	16[111]14[112]BLK	TGTAGCCATTAAAATTCGCATTAAATGCCGGA
G5	13[128]15[127]BLK	GAGACAGCTAGCTGATAAATTAATTTTTGT
H5	13[160]14[144]BLK	GTAATAAGTTAGGCAGAGGCATTTATGATATT
I5	16[143]15[159]BLK	GCCATCAAGCTCATTTTTTAACCACAAATCCA
J5	16[175]14[176]BLK	TATAACTAACAAAGAACGCGAGAACGCCAA
K5	13[192]15[191]BLK	GTAAAGTAATCGCCATATTTAACAAAACTTTT
L5	16[207]14[208]BLK	ACCTTTTTATTTTAGTTAATTTCATAGGGCTT
M5	13[224]15[223]BLK	ACAACATGCCAACGCTCAACAGTCTTCTGA
N5	16[239]14[240]BLK	GAATTTATTTAATGGTTTGAAATATTCTTACC
O5	13[256]15[255]BLK	GTTTATCAATATGCGTTATACAAACCGACCGT
P5	16[271]14[272]BLK	CTTAGATTTAAGGCGTTAAATAAAGCCTGT
A6	11[32]13[31]BLK	AACAGTTTTGTACCAAAAACATTTTATTTC
B6	14[47]12[48]BLK	AACAAGAGGGATAAAAATTTTTAGCATAAAGC
C6	11[64]13[63]BLK	GATTTAGTCAATAAAGCCTCAGAGAACCCTCA
D6	14[79]12[80]BLK	GCTATCAGAAATGCAATGCCTGAATTAGCA
E6	11[96]13[95]BLK	AATGGTCAACAGGCAAGGCAAAGAGTAATGTG
F6	14[111]12[112]BLK	GAGGGTAGGATTCAAAAGGGTGAGACATCCAA
G6	11[128]13[127]BLK	TTTGGGGATAGTAGTAGCATTAAAAGGCCG
H6	11[160]12[144]BLK	CCAATAGCTCATCGTAGGAATCATGGCATCAA
I6	14[143]13[159]BLK	CAACCGTTTCAAATCACCATCAATTCGAGCCA

J6	14[175]12[176]BLK	CATGTAATAGAATATAAAGTACCAAGCCGT
K6	11[192]13[191]BLK	TATCCGGTCTCATCGAGAACAAGCGACAAAAG
L6	14[207]12[208]BLK	AATTGAGAATTCTGTCCAGACGACTAAACCAA
M6	11[224]13[223]BLK	GCGAACCTCCAAGAACGGGTATGACAATAA
N6	14[239]12[240]BLK	AGTATAAAGTTCAGCTAATGCAGATGTCTTTC
O6	11[256]13[255]BLK	GCCTTAAACCAATCAATAATCGGCACGCGCCT
P6	14[271]12[272]BLK	TTAGTATCACAATAGATAAGTCCACGAGCA
A7	9[32]11[31]BLK	TTTACCCCAACATGTTTTAAATTTCCATAT
B7	12[47]10[48]BLK	TAAATCGGGATTCCCAATTCTGCGATATAATG
C7	9[64]11[63]BLK	CGGATTGCAGAGCTTAATTGCTGAAACGAGTA
D7	12[79]10[80]BLK	AAATTAAGTTGACCATTAGATACTTTTGCG
E7	9[96]11[95]BLK	CGAAAGACTTTGATAAGAGGTCATATTTCGCA
F7	12[111]10[112]BLK	TAAATCATATAACCTGTTTAGCTAACCTTTAA
G7	9[128]11[127]BLK	GCTTCAATCAGGATTAGAGAGTTATTTTCA
H7	9[160]10[144]BLK	AGAGAGAAAAAAATGAAAATAGCAAGCAAACT
I7	12[143]11[159]BLK	TTCTACTACGCGAGCTGAAAAGGTTACCGCGC
J7	12[175]10[176]BLK	TTTTATTTAAGCAAATCAGATATTTTTTGT
K7	9[192]11[191]BLK	TTAGACGGCCAAATAAGAAACGATAGAAGGCT
L7	12[207]10[208]BLK	GTACCGCAATTCTAAGAACGCGAGTATTATTT
M7	9[224]11[223]BLK	AAAGTCACAAAATAAACAGCCAGCGTTTTA
N7	12[239]10[240]BLK	CTTATCATTCCCGACTTGCGGGAGCCTAATTT
O7	9[256]11[255]BLK	GAGAGATAGAGCGTCTTTCCAGAGGTTTTGAA
P7	12[271]10[272]BLK	TGTAGAAATCAAGATTAGTTGCTCTTACCA
A8	7[32]9[31]BLK	TTTAGGACAAATGCTTTAAACAATCAGGTC
B8	10[47]8[48]BLK	CTGTAGCTTGACTATTATAGTCAGTTCATTGA
C8	7[56]9[63]BLK	ATGCAGATACATAACGGGAATCGTCATAAATAAAGCAAAG
D8	10[79]8[80]BLK	GATGGCTTATCAAAAAGATTAAGAGCGTCC
E8	7[96]9[95]BLK	TAAGAGCAAATGTTTAGACTGGATAGGAAGCC
F8	10[111]8[112]BLK	TTGCTCCTTTCAAATATCGCGTTTGAGGGGGT
G8	7[120]9[127]BLK	CGTTTACCAGACGACAAAGAAGTTTTGCCATAATTCGA
H8	7[160]8[144]BLK	TTATTACGAAGAACTGGCATGATTGCGAGAGG
I8	10[143]9[159]BLK	CCAACAGGAGCGAACCAGACCGGAGCCTTTAC
J8	10[175]8[176]BLK	TTAACGTCTAACATAAAAACAGGTAACGGA
K8	7[184]9[191]BLK	CGTAGAAAATACATACCGAGGAAACGCAATAAGAAGCGCA
L8	10[207]8[208]BLK	ATCCCAATGAGAATTAACTGAACAGTTACCAG
M8	7[224]9[223]BLK	AACGCAAAGATAGCCGAACAAACCCTGAAC
N8	10[239]8[240]BLK	GCCAGTTAGAGGGTAATTGAGCGCTTTAAGAA
O8	7[248]9[255]BLK	GTTTATTTTGTCACAATCTTACCGAAGCCCTTTAATATCA

P8	10[271]8[272]BLK	ACGCTAACACCCACAAGAATTGAAAATAGC
A9	5[32]7[31]BLK	CATCAAGTAAAACGAACTAACGAGTTGAGA
B9	8[47]6[48]BLK	ATCCCCCTATACCACATTCAACTAGAAAAATC
D9	8[79]6[80]BLK	AATACTGCCCAAAAGGAATTACGTGGCTCA
E9	5[96]7[95]BLK	TCATTCAGATGCGATTTTAAGAACAGGCATAG
F9	8[111]6[112]BLK	AATAGTAAACACTATCATAACCCTCATTGTGA
H9	5[160]6[144]BLK	GCAAGGCCTCACCAGTAGCACCATGGGCTTGA
I9	8[143]7[159]BLK	CTTTTGCAGATAAAAACCAAAATAAAGACTCC
J9	8[175]6[176]BLK	ATACCCAACAGTATGTTAGCAAATTAGAGC
L9	8[207]6[208]BLK	AAGGAAACATAAAGGTGGCAACATTATCACCG
M9	5[224]7[223]BLK	TCAAGTTTCATTAAAGGTGAATATAAAAGA
N9	8[239]6[240]BLK	AAGTAAGCAGACACCACGGAATAATATTGACG
P9	8[271]6[272]BLK	AATAGCTATCAATAGAAAATTCAACATTCA
A10	3[32]5[31]BLK	AATACGTTTGAAAGAGGACAGACTGACCTT
B10	6[47]4[48]BLK	TACGTTAAAGTAATCTTGACAAGAACCGAACT
D10	6[79]4[80]BLK	TTATACCACCAAATCAACGTAACGAACGAG
E10	3[96]5[95]BLK	ACACTCATCCATGTTACTTAGCCGAAAGCTGC
F10	6[111]4[112]BLK	ATTACCTTTGAATAAGGCTTGCCCAAATCCGC
H10	3[160]4[144]BLK	TTGACAGGCCACCACCAGAGCCGCGATTTGTA
I10	6[143]5[159]BLK	GATGGTTTGAACGAGTAGTAAATTTACCATTA
J10	6[175]4[176]BLK	CAGCAAAAGGAAACGTCACCAATGAGCCGC
L10	6[207]4[208]BLK	TCACCGACGCACCGTAATCAGTAGCAGAACCG
M10	3[224]5[223]BLK	TTAAAGCCAGAGCCGCCACCCTCGACAGAA
N10	6[239]4[240]BLK	GAAATTATTGCCTTTAGCGTCAGACCGGAACC
P10	6[271]4[272]BLK	ACCGATTGTCGGCATTTTCGGTCATAATCA
A11	1[32]3[31]BLK	AGGCTCCAGAGGCTTTGAGGACACGGGTAA
B11	4[47]2[48]BLK	GACCAACTAATGCCACTACGAAGGGGGTAGCA
C11	1[64]4[64]BLK	TTTATCAGGACAGCATCGGAACGACACCAACCTAAAACGAGGTCAATC
D11	4[79]2[80]BLK	GCGCAGACAAGAGGCAAAAGAATCCCTCAG
E11	1[96]3[95]BLK	AAACAGCTTTTTGCGGGATCGTCAACACTAAA
F11	4[111]2[112]BLK	GACCTGCTCTTTGACCCCCAGCGAGGGAGTTA
G11	1[128]4[128]BLK	TGACAACTCGCTGAGGCTTGCATTATACCAAGCGCGATGATAAA
H11	1[160]2[144]BLK	TTAGGATTGGCTGAGACTCCTCAATAACCGAT
I11	4[143]3[159]BLK	TCATCGCCAACAAAGTACAACGGACGCCAGCA
J11	4[175]2[176]BLK	CACCAGAAAGGTTGAGGCAGGTCATGAAAG
K11	1[192]4[192]BLK	GCGGATAACCTATTATTCTGAAACAGACGATTGGCCTTGAAGAGCCAC
L11	4[207]2[208]BLK	CCACCCTCTATTCACAAACAAATACCTGCCTA
M11	1[224]3[223]BLK	GTATAGCAAACAGTTAATGCCCAATCCTCA

N11	4[239]2[240]BLK	GCCTCCCTCAGAATGGAAAGCGCAGTAACAGT
O11	1[256]4[256]BLK	CAGGAGGTGGGGTCAGTGCCTTGAGTCTCTGAATTTACCGGGAACCAG
P11	4[271]2[272]BLK	AAATCACCTTCCAGTAAGCGTCAGTAATAA
A12	0[47]1[31]BLK	AGAAAGGAACAACTAAAGGAATTCAAAAAAA
B12	2[47]0[48]BLK	ACGGCTACAAAAGGAGCCTTTAATGTGAGAAT
C12	0[79]1[63]BLK	ACAACTTTCAACAGTTTCAGCGGATGTATCGG
D12	2[79]0[80]BLK	CAGCGAAACTTGCTTTCGAGGTGTTGCTAA
E12	0[111]1[95]BLK	TAAATGAATTTTCTGTATGGGATTAATTTCTT
F12	2[111]0[112]BLK	AAGGCCGCTGATACCGATAGTTGCGACGTTAG
G12	0[143]1[127]BLK	TCTAAAGTTTTGTCGTCTTTCCAGCCGACAA
H12	0[175]0[144]BLK	TCCACAGACAGCCCTCATAGTTAGCGTAACGA
I12	2[143]1[159]BLK	ATATTCGGAACCATCGCCCACGCAGAGAAGGA
J12	2[175]0[176]BLK	TATTAAGAAGCGGGGTTTTGCTCGTAGCAT
K12	0[207]1[191]BLK	TCACCAGTACAAACTACAACGCCTAGTACCAG
L12	2[207]0[208]BLK	TTTCGGAAGTGCCGTCGAGAGGGTGAGTTTCG
M12	0[239]1[223]BLK	AGGAACCCATGTACCGTAACACTTGATATAA
N12	2[239]0[240]BLK	GCCCGTATCCGGAATAGGTGTATCAGCCCAAT
O12	0[271]1[255]BLK	CCACCCTCATTTTCAGGGATAGCAACCGTACT
P12	2[271]0[272]BLK	GTTTTAACTTAGTACCGCCACCCAGAGCCA

Supplementary Table 7 | List of biotinylated staples

No	Pos	Name	Sequence	Mod
1	C02	18[63]20[56]BIOTIN	ATTAAGTTTACCGAGCTCGAATTCGGGAAACCTGTCGTGC	5'-BT
2	C09	4[63]6[56]BIOTIN	ATAAGGGAACCGGATATTCATTACGTCAGGACGTTGGGAA	5'-BT
3	G02	18[127]20[120]BIOTIN	GCGATCGGCAATTCCACACAACAGGTGCCTAATGAGTG	5'-BT
4	G09	4[127]6[120]BIOTIN	TTGTGTCGTGACGAGAAACACCAAATTTCAACTTTAAT	5'-BT
5	K02	18[191]20[184]BIOTIN	ATTCATTTTTGTTTGGATTATACTAAGAAACCACCAGAAG	5'-BT
6	K09	4[191]6[184]BIOTIN	CACCCTCAGAAACCATCGATAGCATTGAGCCATTTGGGAA	5'-BT
7	O02	18[255]20[248]BIOTIN	AACAATAACGTAAAACAGAAATAAAAATCCTTTGCCCGAA	5'-BT
8	O09	4[255]6[248]BIOTIN	AGCCACCACTGTAGCGCGTTTTCAAGGGAGGGAAGGTAAA	5'-BT
